# Heterogeneous Myocardial Adaptation to Pressure Overload Reveals a Spared, Vulnerable Segment Identified by Multiparametric Cardiac MRI

**DOI:** 10.1002/nbm.70288

**Published:** 2026-04-09

**Authors:** Vitali Koch, Andreas T. Ochs, Simon Martin, Thomas Vogl, Marco M. Ochs, David M. Leistner, Sebastian M. Haberkorn

**Affiliations:** ^1^ Goethe University Frankfurt, University Hospital Department of Radiology Frankfurt Germany; ^2^ Goethe University Frankfurt, University Hospital Department of Cardiology and Angiology Frankfurt Germany; ^3^ German Center of Cardiovascular Research (DZHK) Partner Site Rhine/Main Frankfurt Germany; ^4^ Cardiopulmonary Institute (CPI) Frankfurt Germany

**Keywords:** cardiac hypertrophy, cardiac MRI, myocardial relaxation times, myocardial tissue characterization, quantitative imaging, spared hypertrophy, T1 mapping, T2 mapping

## Abstract

Cardiac adaptation to chronic pressure overload is traditionally viewed as a uniform concentric hypertrophy; however, growing evidence suggests that the myocardium responds in a spatially heterogeneous manner. This study aimed to systematically map regional and temporal adaptation patterns of the left ventricle (LV) under experimental pressure overload using high‐resolution multiparametric cardiac MRI, with the hypothesis that distinct regional adaptation phenotypes contribute to both local functional differences and global ventricular dysfunction. Thirty‐seven male 129/SvEv mice underwent transverse aortic constriction (TAC) surgery and were followed longitudinally over 7 weeks. Cine imaging, T1‐ and T2‐mapping were performed at baseline, 3 days, 4 weeks and 7 weeks using a 9.4 T small‐animal scanner. A 200‐sector LV segmentation enabled quantitative regional analysis of wall thickness, fractional shortening (FS) and relaxation times. Global and regional imaging parameters were correlated with histological and biochemical indices at study termination, and statistical analyses were performed using the Mann–Whitney test and correlation modelling, with *p* < 0.05 considered significant. All TAC animals developed global LV hypertrophy and an early increase in LV mass (+62% ± 15% at Week 7, *p* < 0.001). Unexpectedly, in 18 animals (49%), a discrete basolateral LV segment failed to hypertrophy (spared hypertrophy, SH) despite uniform pressure elevation. This region exhibited markedly reduced regional FS (10.2% ± 3.1% vs. 24.9% ± 2.2%, *p* = 0.01) and was associated with lower global ejection fraction (32.5% ± 1.8% vs. 49.8% ± 4.1%, *p* < 0.05) and LV dilatation. Parametric MRI revealed distinct tissue signatures: T1 increased in hypertrophied sectors (+22.6% ± 1.1%), whereas it decreased in SH sectors (−5.1% ± 1.7%, *p* < 0.01) over the 7‐week observation period. Sector‐wise correlations linked T1 shortening with reduced FS (*R* ^2^ = 0.58, *p* = 0.002), suggesting maladaptive regional remodeling. In conclusion, pressure overload elicits diverse regional adaptation, and failure to hypertrophy identifies myocardial territories prone to early dysfunction and global decline. Recognizing hypertrophy as an essential adaptive mechanism shifts the paradigm of pressure‐overload remodeling. Multiparametric MRI provides a translational platform for detecting regional vulnerability and guiding early intervention.

## Graphical abstract

Graphical summary of a longitudinal multiparametric MRI study in mouse TAC model, showing imaging at baseline, 3 days, 4 weeks and 7 weeks. All TAC animals developed global LV hypertrophy (+62% LV mass at week 7), but half displayed a spared‐hypertrophy basolateral region. This non‐adapting sector showed reduced fractional shortening, lower ejection fraction, LV dilatation, decreased T1, contrasting with elevated T1 in hypertrophied myocardium. The schematic highlights uniform versus spared hypertrophy, linking regional adaptation failure to early dysfunction global decline.

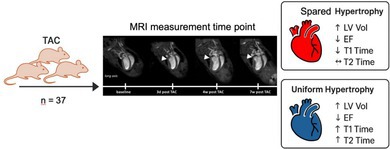

**Abbreviations**
CA: contrast agent
CMR: cardiovascular magnetic resonance
dWT: diastolic wall thickness
EF: ejection fraction
FS: fractional shortening
LV: left ventricle
TAC: transverse aortic constriction
SH: spared hypertrophy

## 1 Introduction

Cardiac structural and functional adaptation to increased pressure load is a fundamental challenge to the myocardium that underpins a variety of clinical pathologies, yet the spatial and temporal heterogeneity of these adaptations remains incompletely characterised [1]. In humans, chronic pressure overload—such as that arising from aortic stenosis or systemic hypertension—drives left ventricular (LV) hypertrophy and eventually precipitates heart failure if compensatory mechanisms are exhausted [2, 3]. Experimental animal models have been instrumental in dissecting underlying pathophysiological mechanisms, but many such studies focus on global hypertrophic responses rather than the detailed phenotypic heterogeneity of regional myocardial adaptation [4]. For example, recent reviews emphasise that adaptations of wall stress, extracellular matrix remodelling and cardiomyocyte growth differ both spatially within the ventricle and temporally during progression from compensation to decompensation [5].

Moreover, it is increasingly recognised that adaptation is not uniform across myocardial regions: variations in hypertrophy severity, fibre architecture, perfusion and metabolic shifts may contribute to regional differences in contractile performance and ultimately global ventricular dysfunction [6]. For instance, experimental models of pressure overload demonstrate that not only cardiomyocyte hypertrophy but also immune‐cell infiltration, fibroblast activation and extracellular matrix deposition vary regionally, influencing regional stiffness and contractile reserve [7]. In turn, computational growth‐and‐remodelling frameworks suggest that local stress–strain heterogeneity may drive differential hypertrophic and fibrotic responses across the myocardium [8].

Against this background, the use of advanced imaging methods offers a compelling opportunity to interrogate myocardial adaptation with high spatial resolution and temporal tracking [9, 10]. Consequently, deeper insight into how specific myocardial regions respond and adapt to pressure overload could refine understanding of the transition from early compensation to maladaptive remodelling and heart failure.

In this context, our study aimed to phenotypically characterise the heterogeneity of adaptation mechanisms in response to experimentally increased pressure overload in a well‐controlled animal model. Specifically, we set out to systematically investigate spatial and temporal changes in myocardial morphology, function, tissue composition and metabolic signatures under experimental pressure elevation, in order to test the hypothesis that distinct myocardial regions exhibit divergent adaptive trajectories, rather than a homogeneous hypertrophic reaction. We postulate that such heterogeneity may contribute to both preserved global function in early phases and to regional failure‐prone zones in later maladaptive stages. Addressing this hypothesis serves two important objectives: firstly, to delineate the regional complexity of adaptation beyond bulk measurements of hypertrophy and function; secondly, to identify phenotypic signatures that may predict which regions become vulnerability sites for eventual dysfunction.

To fulfil these objectives, we applied a multi‐modal approach combining controlled pressure elevation, magnetic resonance‐based assessments, histology and tissue metabolomics in a time‐series design. By mapping adaptation patterns across the myocardium and over time, we sought to define key mechanistic pathways underpinning regional adaptation and to relate these to functional outcomes. We anticipate that the resulting dataset will not only deepen mechanistic understanding but also provide a translational bridge towards identifying early imaging biomarkers of region‐specific vulnerability in pressure‐overload cardiomyopathy.

In summary, the aim of our investigation is to uncover and characterise myocardial adaptation heterogeneity under experimental pressure overload, to identify phenotypic regional signatures of adaptation (or maladaptation) and to link these signatures to ventricular function. We believe that this work can deliver novel insights into tissue responses using magnetic resonance‐based methodologies and to advance biomedical understanding of disease processes.

## 2 Methods

This study aimed to systematically characterize spatial and temporal patterns of myocardial adaptation to experimental pressure overload in an animal model, using high‐resolution multiparametric cardiac MRI to identify distinct regional phenotypes and mechanisms of hypertrophic remodeling. All animal studies have been approved by the *Landesamt für Natur‐ und Umweltschutz (LANUV)* by the registration number 84‐02.04‐2015‐A089 in accordance with the ethical standards laid down in the 1964 Declaration of Helsinki and its later amendments.

### 2.1 Animal Experiments

All animal experiments were performed in accordance with the national guidelines of animal care. 37 male wild type 129/SvEv mice (20–29 g body weight, 8–16 weeks of age) were used in this study and bred at the central animal facilities. The planned sample size was based on previously published TAC studies using cardiac magnetic resonance imaging and comparable functional endpoints. Assuming an approximately equal (50/50) separation between outcome groups, a total of 40 animals was targeted to ensure adequate group sizes for statistical comparison. Three animals were excluded due to intraoperative mortality (*n* = 2) or unsuccessful TAC caused by postoperative suture dehiscence (*n* = 1), resulting in a final study population of 37 mice. They were fed with a standard chow diet and had continuously access to tap water. For introduction of hypertrophy, mice were anaesthetized with isoflurane (1.5%), analgised with 0.1 mg/kg Buprenorphine and intubated for mechanical ventilation. Following partial thoracotomy, the transverse aorta was exposed between the brachiocephalic trunk and the left common carotid artery. The aorta was then ligated using a 7–0 silk suture against a 27‐gauge needle, which was subsequently removed to create a standardized stenosis. The chest was closed and animals were extubated after recovery of spontaneous breathing [11]. The adequacy of the constriction was verified by transthoracic echocardiography using pulsed‐wave Doppler measurements at the site of the ligature to determine peak blood flow velocity and the resulting pressure gradient across the stenosis [11].

**FIGURE 1:**
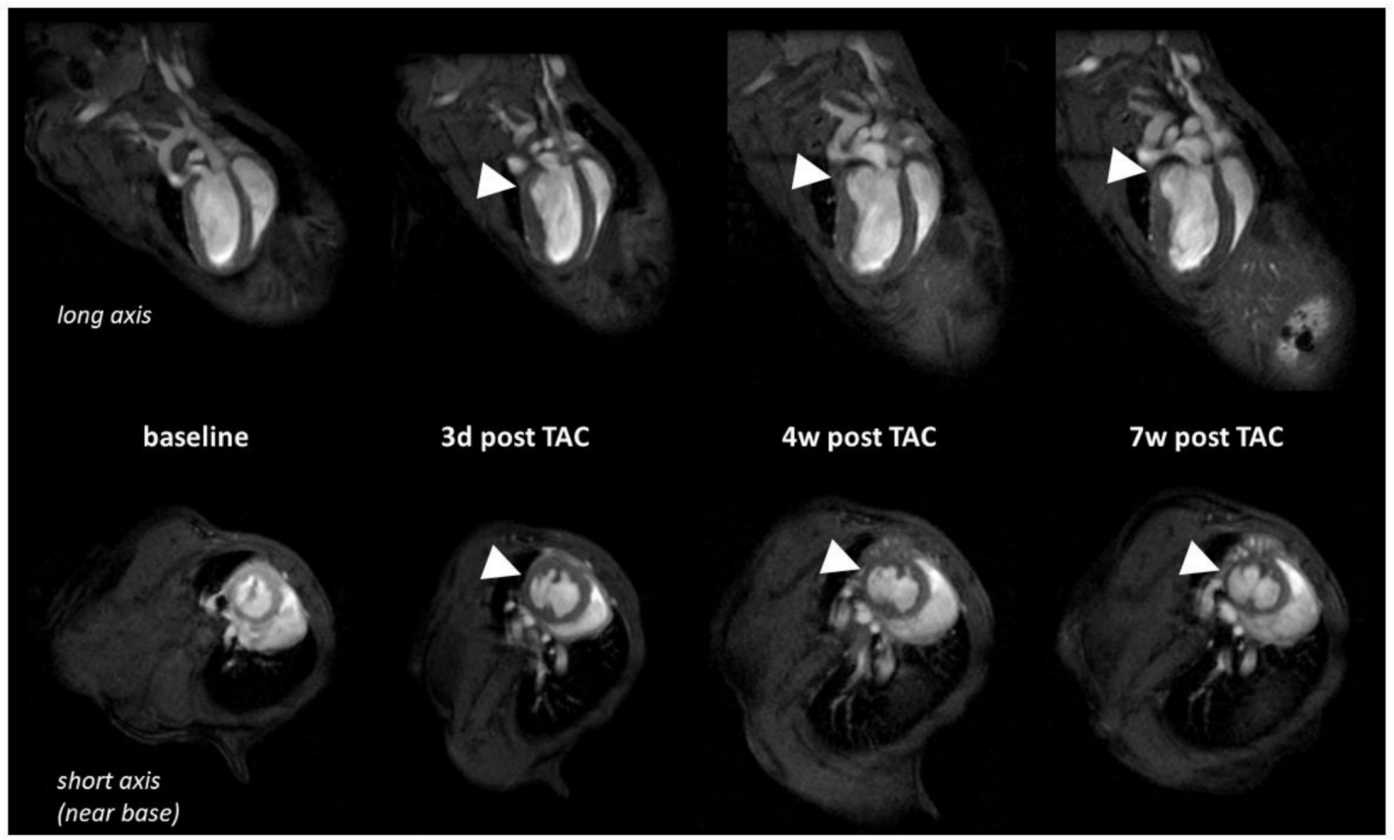
Top: Long axis slices through the LV in enddiastole at baseline, 4 days, 4 weeks and 7 weeks after TAC in one mouse with SH (arrow tip) in the LV basolateral wall. Bottom: Corresponding enddiastolic short axis slices at the basal level of the LV. Regions of SH are indicated by arrow tips. LV left ventricle, TAC Transverse aortic constriction, SH Spared hypertrophy.

After the mice were subjected to transverse aortic constriction (TAC), we monitored them by comprehensive CMR over a period of 7 weeks (Figure 1). Postoperative pain management was maintained with repeated subcutaneous administration of buprenorphine (0.05–0.1 mg/kg) at regular intervals during the first 48 h after surgery to ensure adequate analgesia. Animals were monitored closely for signs of postoperative pain or distress, and additional doses were administered if required according to institutional animal welfare guidelines.

### 2.2 Magnetic Resonance Imaging Studies

MRI data recording was performed on a Bruker AVANCE III 9.4 T wide bore NMR spectrometer (Bruker, Rheinstetten, Germany) at 400.13 MHz operated by ParaVision 5.1. Acquisition of images was done using the Bruker Microimaging unit Micro 2.5 with actively shielded gradient sets and a 25 mm birdcage resonator.

Mice were anaesthetized with 1.5% isoflurane and kept at 39°C to prevent anesthesia‐associated hypothermia. The heating system was set to approximately 39°C to counteract heat loss during prolonged anesthesia. This setting does not necessarily reflect the animals' core body temperature but was used to maintain physiological normothermia throughout the experiment. ECG and respiration was monitored through electrodes (Klear‐Trace; CAS Medical Systems, Branford) and a pneumatic pillow. The vital function data were processed by a M1025 system (SA Instruments, Stony Brook, NY, USA) and used to synchronize the MRI data acquisition with cardiac and respiratory motion.

In our multiparametric MR analysis, first ECG‐ and respiratory‐triggered high resolution cine movies were recorded in short and long axis orientation covering the entire LV for detailed analysis as previously described [12]. In connection, native T1 and T2 maps in short axis orientation at the level of the LV base were acquired. As previously published by our group, T2 maps were acquired with a gated multi‐echo sequence (16 echoes, separated by a TE = 5 ms; TR = 500 ms, TA ≈2 min, ST = 1 mm, FOV = 30 × 30 mm^2^, MS = 128 × 128, NS = 2) [13]. We only used one early ECG trigger to maintain constant conditions for the recording of the echo train. An exponential decay curve was fitted to the intensity decline of each pixel within the images obtained from the 16 echoes in order to create the T2 maps. For quantification of T1 values we used a different approach, first introduced by Coolen et al. with a retrospectively triggered *fast low angle shot* sequence (IntragateFLASH) and variable flip‐angle analysis [14]. Short echo times (TE = 1.26 ms) and in particular constant repetition times (TR = 5.82 ms) allowed us the generation of high quality, artefact‐free T1 maps with excellent spatial resolution (in plane 100 × 100 μm^2^).

The scanning protocol took around 40 min and was well tolerated by all animals, which recovered from anaesthesia within 2 min after the experiment.

### 2.3 Hypothesis and Endpoints

The primary hypothesis of this study was that global LV hypertrophy represents the physiological adaptive response to increased afterload following TAC. Accordingly, the primary endpoints were changes in LV mass, global LV function and left LV end‐diastolic volume. Secondary endpoints included regional fractional shortening (FS) and structural parameters reflecting maladaptive remodeling, specifically regional diastolic wall thickness (dWT). Additional parameters, including native T1 and T2 relaxation times and histological findings, were analysed as exploratory endpoints to characterize associated tissue alterations.

### 2.4 Image Analysis and Regional Quantification

**FIGURE 2:**
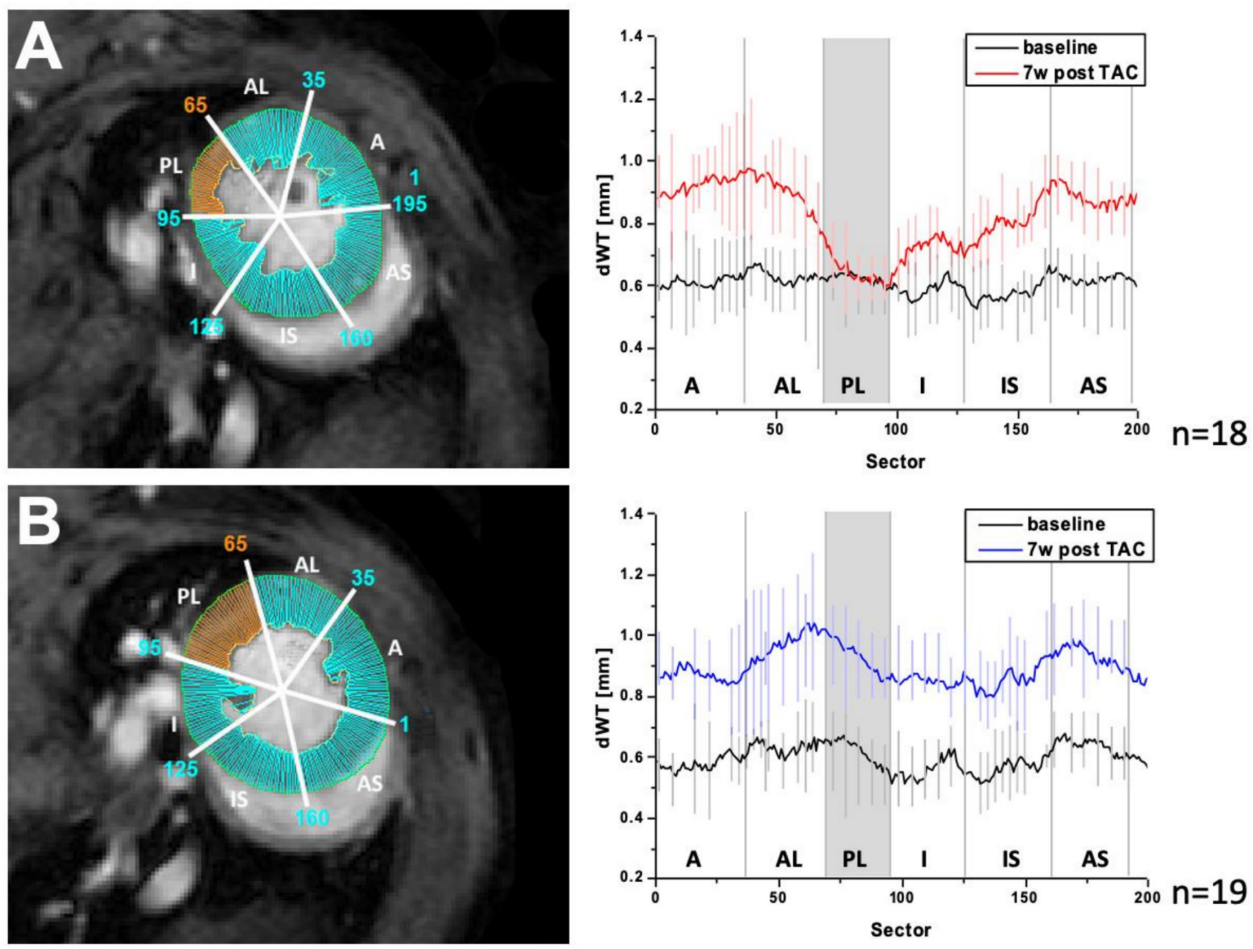
(A) Short axis slice at the basal level of the LV in diastole 7 weeks post TAC in an animal with SH. dWT analysis over 200 sectors of the LV is indicated by the cyan marks. The SH in the posterolateral segment is highlighted in orange. Mean dWT measures of all 18 mice with SH are displayed with SD at baseline (black) and 7 weeks post TAC (red) showing a SH exclusively in the posterolateral segment, whereas a distinct hypertrophy is seen in all other sectors of the LV. (B) Short axis slice at the basal level of the LV in diastole 7 weeks post TAC in an animal without SH. dWT analysis over 200 sectors of the LV is indicated by the cyan marks. The posterolateral segment is highlighted in orange. Mean dWT measures of all 19 mice without the occurrence of SH with SD over the mentioned 200 sectors are displayed at baseline (black) and 7 weeks post TAC (blue) indicating an equally contributed hypertrophy over the entire LV dWT diastolic wall thickness, LV left ventricle, TAC Transverse aortic constriction, SH Spared hypertrophy.

For regional characterization all data sets were analysed over 200 sectors and FS as well as dWT were calculated for each sector (Figure 2).

**FIGURE 3:**
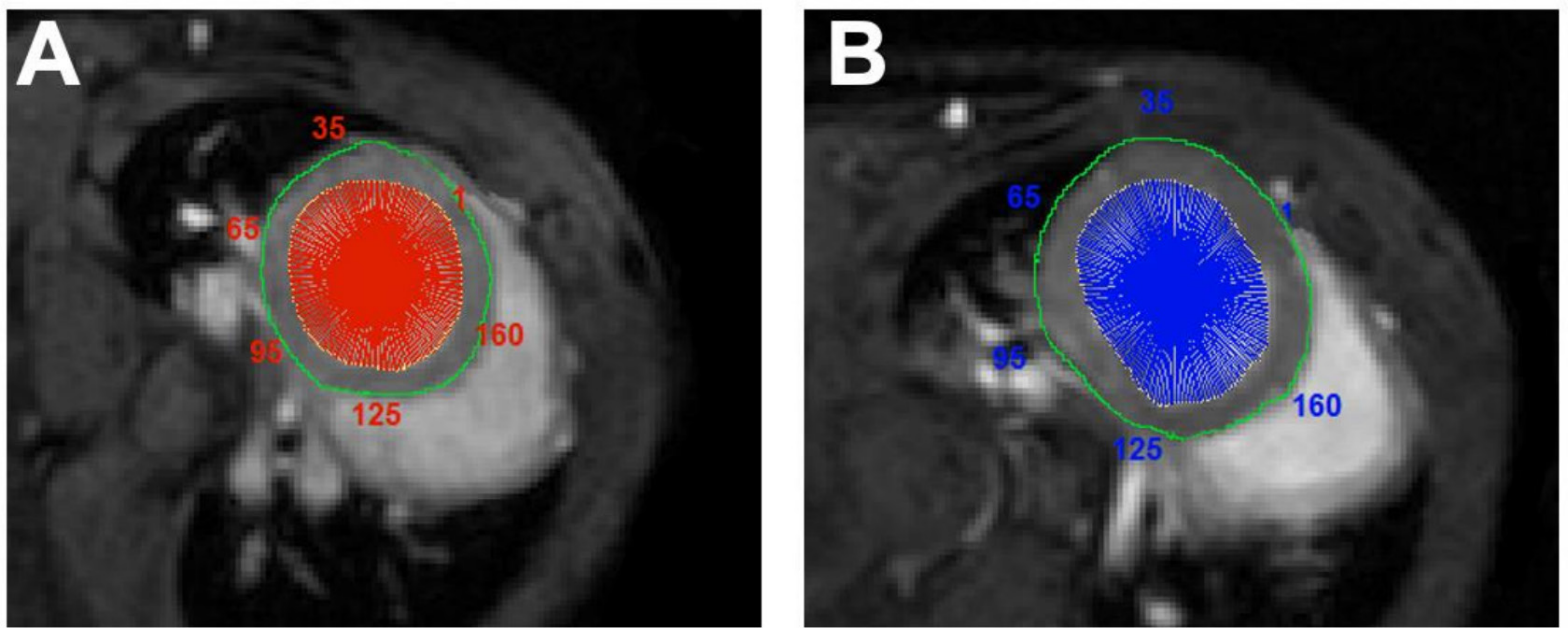
(A) Short axis slice at the basal Level of the heart in an animal where we observed a SH after TAC procedure. (B) Short axis slice at the basal Level of the heart in an animal where we did not observe a SH after TAC procedure. TAC Transverse aortic constriction, SH Spared hypertrophy.

**FIGURE 4:**
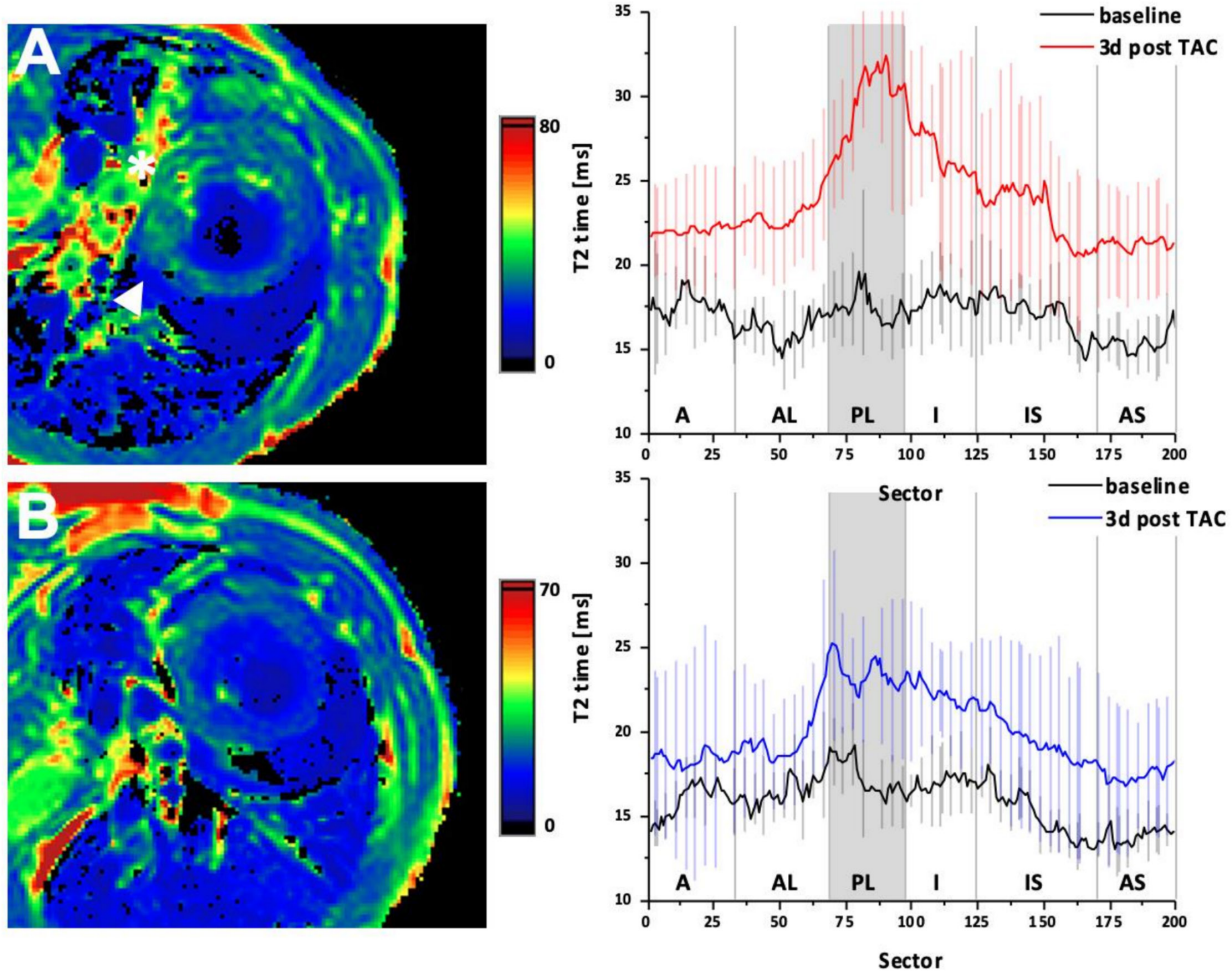
(A) T2 Map short axis slice at the basal level of the LV 3 days post TAC in an animal without SH. T2 relaxation times are significantly elevated in the PL area (star) as well as in the surrounding sectors (arrow tip) of the LV, indicated by a greenish tint in the color‐coded map. Mean T2 measures of all 18 mice with the occurrence of SH over the mentioned 200 sectors are displayed at baseline (black) and 3 days post TAC (red) including SD. The posterolateral segment is highlighted in gray, where relaxation times are highest. (B) T2 Map short axis slice at the basal level of the LV 3 days post TAC in an animal without SH. T2 relaxation times are slightly elevated in the posterolateral and the surrounding areas of the LV, which is indicated by the green tint. Mean T2 measures of all 19 control mice are displayed with SD at baseline (black) and 3 days post TAC (blue) showing a moderate oedema in the posterolateral segment and the surrounding area. LV left ventricle, TAC Transverse aortic constriction, SH Spared hypertrophy.

Multiparametric MRI data were analysed in short axis orientation at the basal level of the LV involving the side of SH. Ventricular demarcations in diastolic and systolic phases were manually drawn with the ParaVision region‐of‐interest (ROI) tool (Bruker, Rheinstetten, Germany) as previously described [13]. Ejection fraction (EF) was calculated by the known formula (EDV—ESV/EDV). Since global MRI values can be unaffected by regional hypercompensation, we rather decided to analyse our data from a regional perspective to overcome these bias problems. We used in‐house developed software based on LabVIEW (National Instruments, Austin, USA) for a sectorial analysis of the LV, which was systematically divided into 200 equivalent sectors starting from the upper insertion point of the right ventricle as previously described (Figure 3) [13]. This provided us with the possibility to locally compare all measures of our multiparametric MRI analysis in the anatomically corresponding areas and at different time points. Values of sectoral FS were calculated as the difference of the radii measured from the centre of mass in end‐diastole and end‐systole (Figure 2), divided by the end‐diastolic value, analogous to the M‐mode‐derived FS in echocardiography. Briefly, a central reference point was defined in the LV lumen and kept constant between end‐diastole and end‐systole. However, the M‐mode‐derived FS, in contrast, is solely derived from two opposing regions of the heart, whereas here we used 200 radial approaches. The same sectoral definitions were applied for the analysis of the relaxometric maps (Figures 4 and 7), where the T1 respectively T2 relaxation times were averaged over the myocardial layers in each of the 200 myocardial sectors within the lines pointing from the centre of the cavity.

### 2.5 Histology and Biochemistry

Immediately after the final imaging time point (7 weeks), animals were euthanised with an overdose of pentobarbital (> 100 mg/kg) and the hearts excised, rinsed in ice‐cold saline, weighed and sectioned at the mid‐ventricular level [15]. One slice was fixed in 10% formalin, embedded in paraffin and stained with haematoxylin–eosin and picrosirius red for quantification of cardiomyocyte cross‐sectional area (CSA) and interstitial collagen fraction (ICF) [15]. These approaches permit correlation of in vivo MRI parameters with ex vivo phenotypic signatures of adaptation [15] (Figure 6).

### 2.6 Statistical Analysis

Unless otherwise indicated, all values are given as the mean ± standard deviations (SD). All statistical analysis was performed using a statistical software package (GraphPad Prism 5, La Jolla, USA). Data were statistically analysed by Mann–Whitney test and unpaired Student's *t* test. Coefficient of determination was done by adjusted R square. *p* values below 0.05 were assumed to be significant.

## 3 Results

### 3.1 Global Haemodynamic and Morphological Adaptation

**FIGURE 5:**
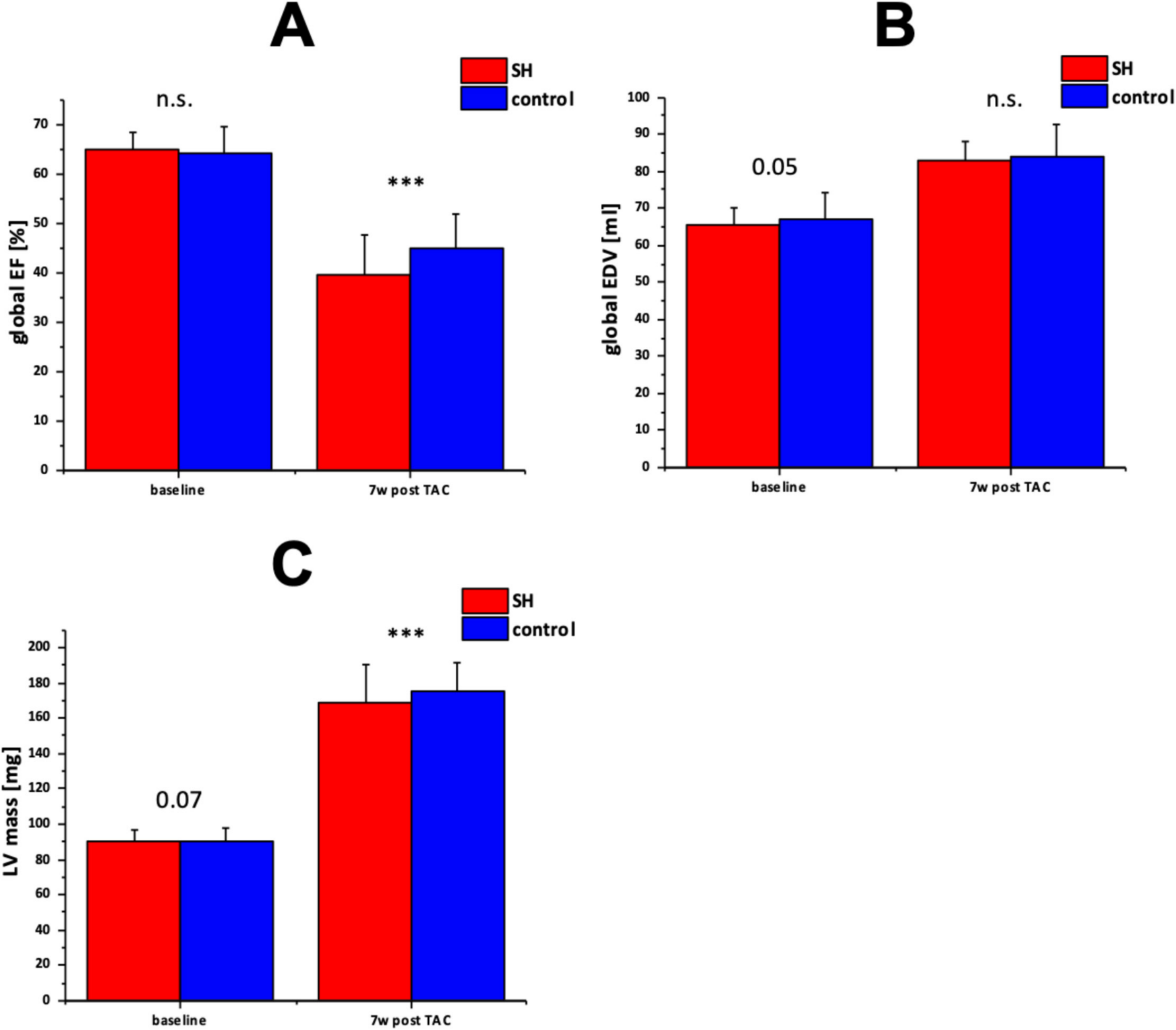
Global values of mice with (red) and without (blue) the occurrence of a SH after TAC operation. (A) Mean vales with SD of global 3D EF in SH animals (red) decreases significantly more as compared to the pseudo control (blue) group at 7 weeks post TAC. (B) Mean EDV with SD in the represented groups do not differ prior or past the intervention. (C) LV mass of mice with SH is significantly lower than in control animals. EDV enddiastolic volume, LV left ventricle, TAC Transverse aortic constriction, SH Spared hypertrophy.

All animals subjected to experimental pressure elevation completed the imaging protocol without premature loss. At the first post‐intervention time point (Day 3) we confirmed a significant rise in peak pressure gradient across the imposed stenosis (mean increase 48 ± 7 mmHg compared to baseline; *p* < 0.01), consistent with a reproducible afterload elevation. Over the full 7‐week follow‐up, animals developed pronounced LV hypertrophy: LV mass indexed to body weight increased by 45% ± 12% at Week 4 and 62% ± 15% at Week 7 (both *p* < 0.001 vs. baseline) (Figure 5C). EF remained within normal limits at Day 3 (mean 55.4% ± 3.9%) but declined significantly by Week 7 (41.8% ± 4.2%, *p* < 0.01), indicating emerging functional decompensation (Figure 5A).

### 3.2 Identification of a Spared‐Hypertrophy (SH) Region

**FIGURE 6:**
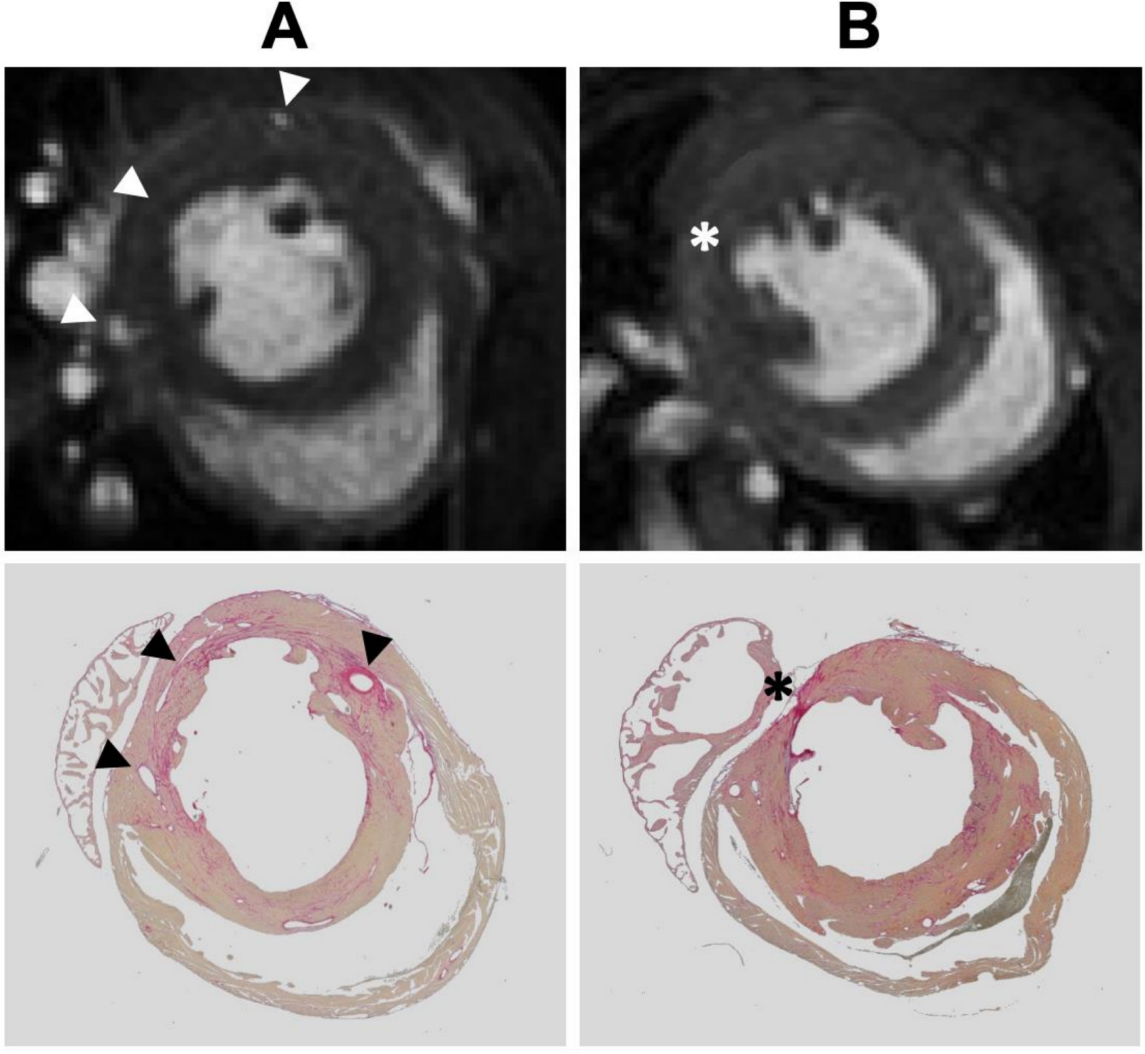
(A) Top: Short axis cine loop in diastole at the LV base in a ‘control’ mouse 7 weeks after TAC. Coronary arteries at the lateral LV side are highlighted by arrow tips. Bottom: Merging anatomy in a Pico Sirius red staining 7 weeks post TAC indicating slight fibrosis over the entire LV. The three identified coronary arteries from the cine loop are highlighted by arrow tips. (B) Top: Short axis cine loop in diastole at the LV base in an animal with SH weeks after TAC. Coronary arteries at the lateral LV side are not visbile. SH ate the posterolateral side is indicated by a *star*. *Bottom* Merging anatomy in a Pico Sirius red staining 7 weeks post TAC indicating severe fibrosis at the posterolateral side of the LV. Here the otherwise clearly visbile hypertrophy is spared (star). LV left ventricle, TAC Transverse aortic constriction, SH Spared hypertrophy.

Unexpectedly, detailed regional analysis revealed that in 18 of our 37 animals (49%) a defined LV segment—the basolateral segment at the level of the LV base—exhibited minimal increase in wall thickness despite global hypertrophy (Figure 1). We designate this region as the spared hypertrophy (SH) segment. In these animals, the dWT within the affected sector at Week 7 increased only by 9.2% ± 10.7% (dWT 0.64 mm [95% CI: 0.61; 0.68]) compared to baseline, whereas in animals without such regional sparing the corresponding sectors exhibited an increase of 47.3% ± 18.9% (dWT 0.88 mm [95% CI: 0.83; 0.94]) (*p* < 0.01) (Figure 2A,B) This observation suggests a regional failure of cardiomyocyte hypertrophic growth under pressure overload in a discrete myocardial territory (Figure 6).

### 3.3 Temporal Progression of Regional Hypertrophy and Wall Thickness

In animals without SH, wall thickness rose steadily from baseline (1.8 ± 0.2 mm) to Week 4 (2.4 ± 0.3 mm) and Week 7 (2.6 ± 0.3 mm) (Figure 2). In contrast, in the SH group the basolateral sector exhibited only a modest rise (1.8 ± 0.2 mm → 2.0 ± 0.2 mm at Week 4 → 2.0 ± 0.3 mm at Week 7). The divergence became statistically significant at Week 4 (*p* < 0.05) and remained so at Week 7 (*p* < 0.01). Importantly, other sectors in SH‐animals did hypertrophy similarly to non‐SH animals, indicating that the regional sparing was selective rather than global.

### 3.4 Regional Functional Consequences—FS

To assess the functional implications of regional structural non‐adaptation, we performed sectoral FS analysis across the 200‐sector radial grid. At Week 7, in the posterolateral sectors (LV base) affected by SH the mean FS was 10.2% ± 3.1%, significantly lower than the mean FS in comparable sectors of animals without SH (24.9% ± 2.2%, *p* = 0.01). In well‐hypertrophied sectors (non‐SH sectors) of both groups, FS was similar (26.3% ± 2.8% vs. 25.0% ± 2.4%, n.s.), indicating that hypertrophy per se did not preclude preserved regional contraction. These findings highlight that the presence of a non‐hypertrophied region under elevated load strongly correlates with impaired local systolic performance.

### 3.5 Global Functional and Volumetric Consequences

Beyond the regional impairment, the presence of SH was also associated with adverse global cardiac remodelling. At Week 7, animals with SH had a significantly lower EF (32.5% [95% CI: 31.6%; 33.4%]) compared with animals without SH (49.8% [95% CI: 47.8%; 51.8%], *p* < 0.05) (Figure 5A). In addition, end‐diastolic volumes (EDV) were greater in SH‐animals (110.9 μL [95% CI: 109.4; 112.4 μL]) than in non‐SH animals (89.1 μL [95% CI: 84.2; 94.0 μL], *p* < 0.05) (Figure 5B), consistent with LV dilatation and early maladaptive remodelling. These global changes suggest that regional adaptation failure may impose a burden on overall ventricular performance, shifting the balance from compensation to early decompensation.

### 3.6 Parametric MRI Tissue Characterization—Native T1 and T2 Mapping

**FIGURE 7:**
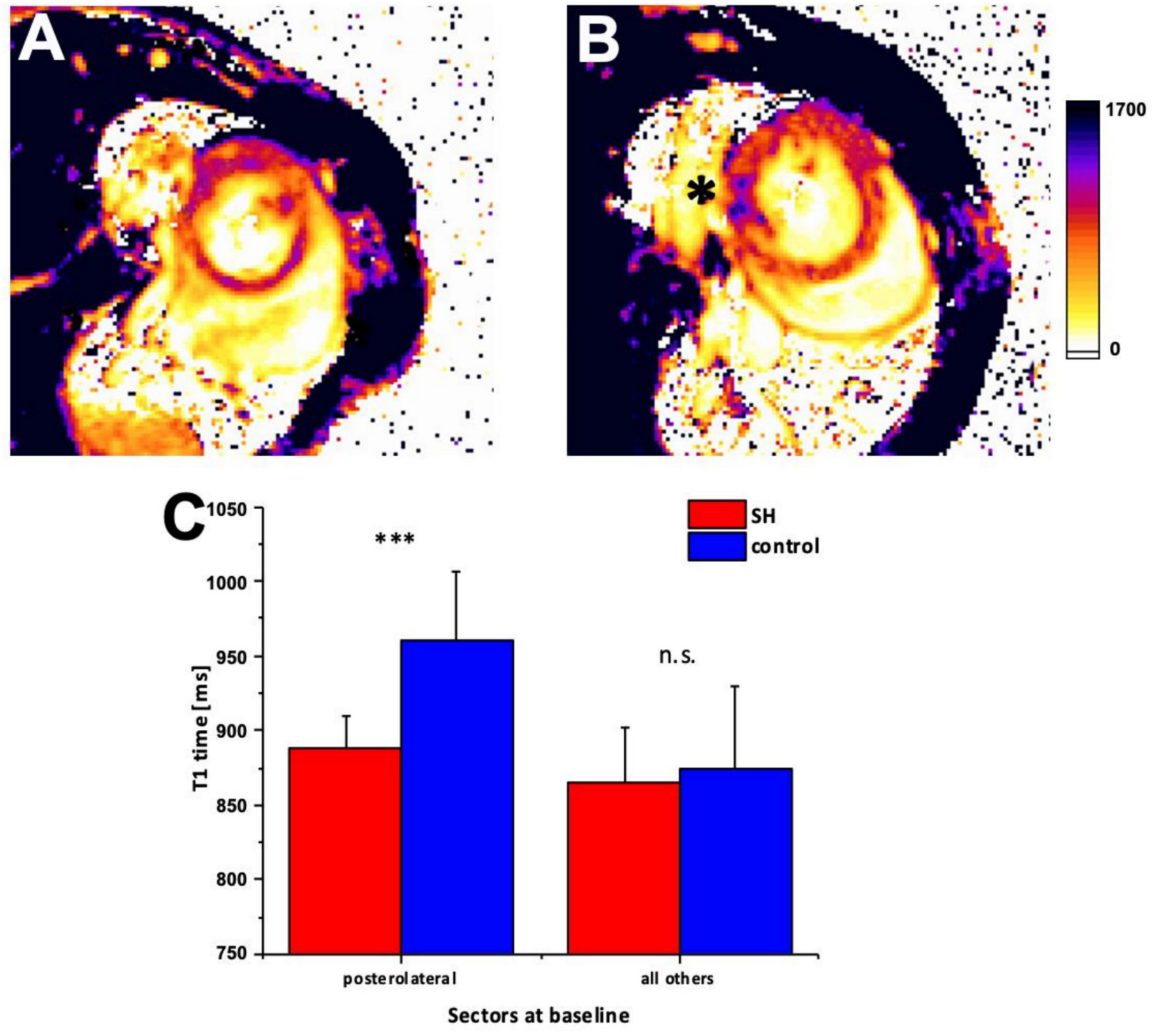
A T1 Map short axis slice at the basal level of the LV before the onset of TAC operation in an animal with SH. T1 relaxation times are equally distributed over all 200 sectors of the LV, indicated by a predominantley orange tint in the color‐coded map. B T1 Map short axis slice at the basal level of the LV before TAC operation in an animal without SH. T1 relaxation times are locally increased in the posterolateral (star) segment of the LV, which is indicated by the purple tint. C Mean T1 measures of all mice are displayed with SD for the PL and for all other sectors, respectively, suggesting a performed abbreviation of the local tissue composition in the PL sector of control animals before the onset of surgery. LV left ventricle, TAC Transverse aortic constriction, SH Spared hypertrophy.

To explore underlying tissue changes in hypertrophy and in the SH region, we acquired native T1 and T2 relaxation maps at each time point. In well‐hypertrophied sectors, native T1 increased by 22.6% ± 1.1% (*p* < 0.01) (876 ms [95% CI: 858; 894 ms] vs. 1074 ms [95% CI: 1062; 1085 ms]) at Week 7 compared to baseline, consistent with hypertrophic wall thickening, increased interstitial expansion and likely collagen deposition (Figure 7A,B). In contrast, in the posterolateral SH sectors of the same hearts, native T1 decreased by 5.1% ± 1.7% (*p* < 0.01 vs. non‐SH sectors) (885 ms [95% CI: 873; 897 ms] vs. 840 ms [95% CI: 826; 855 ms]) (Figure 7C). T2 values showed a modest but statistically significant prolongation in hypertrophied sectors at Week 7 (+11.4% ± 2.3%, *p* < 0.05), whereas SH sectors exhibited no significant T2 change (−1.2% ± 2.0%, NS) (Figure 4). These differential relaxation patterns suggest a fundamentally different tissue phenotype in the SH‐region: despite persistent overload, the region did not mount typical hypertrophic/interstitial responses but instead manifested a unique tissue state characterised by paradoxical T1 shortening and absence of T2 elevation.

### 3.7 Sectoral Correlation Between Structural, Functional and Tissue Parameters

To probe the interrelationship between structure, tissue characterisation and function, we performed sector‐wise correlation analyses across all animals. In hypertrophied sectors, higher dWT correlated strongly with increased native T1 (*R* ^2^ = 0.72, *p* < 0.001) and with preserved regional FS (*R* ^2^ = 0.65, *p* < 0.001). By contrast, in the SH sectors, despite low dWT, native T1 shortening correlated with severely reduced FS (*R* ^2^ = 0.58, *p* = 0.002) and with greater EDV dilatation (*R* ^2^ = 0.49, *p* = 0.004). These data imply that while hypertrophic growth is associated with relatively preserved regional performance, absence of hypertrophy—despite elevated load—is associated with tissue changes that adversely impact contraction and drive global remodelling.

### 3.8 Temporal Evolution of Adaptation Trajectories

In typical hypertrophied sectors, dWT rose steadily (from baseline [1.8 ± 0.2 mm] to Week 7 [2.6 ± 0.3 mm]), FS remained near baseline until Week 4 then modestly declined by Week 7 (26.3% ± 2.8% vs. 25.0% ± 2.4%, n.s.) and T1 rose gradually (22.6% ± 1.1%; *p* < 0.01). In SH sectors, dWT remained flat (1.8 ± 0.2 mm → 2.0 ± 0.2 mm at Week 4 → 2.0 ± 0.3 mm at Week 7), FS began to decline already by Week 4 (−15% ± 4% vs. baseline, *p* < 0.05) and by Week 7 FS was deeply reduced (−60% ± 6%), while T1 first remained stable then dropped at Week 7 (−5.1% ± 1.7%) (*p* < 0.01). Importantly, the time difference between functional decline and global compensation failure in SH‐animals suggests that region‐specific adaptation failure may act as an early driver of maladaptive remodelling (Figure 6).

## 4 Discussion

In this study we set out to examine the phenotypic and spatio‐temporal dynamics of myocardial adaptation in an experimental model of pressure overload, with a particular focus on regional heterogeneity of response. Our principal findings are: (1) despite uniform global afterload elevation, a clearly demarcated myocardial segment (the posterolateral basal sector of the left ventricle) in half of the animals failed to mount hypertrophic growth (the ‘spared hypertrophy’ or SH region); (2) this non‐hypertrophied region was associated with markedly impaired regional contractile performance (reduced FS) and the hearts exhibiting SH displayed significant global systolic dysfunction (reduced ejection fraction) and dilatation (higher end‐diastolic volume); (3) tissue‐characterisation by relaxation mapping (native T1 and T2) revealed a distinct phenotype in the SH region (paradoxical T1 shortening, static T2) compared to hypertrophied sectors (T1 prolongation, T2 elevation), suggesting fundamentally different adaptation trajectories; and (4) sector‐wise correlation analyses indicated that in hypertrophied sectors increased wall thickness correlated with preserved local function, whereas in SH sectors low wall thickness and reduced function were strongly inter‐related. Together, these observations suggest that not only exaggerated hypertrophy but also regional failure to hypertrophy under elevated load may herald functional decline and trigger adverse remodelling.

### 4.1 Summary of Findings

In summary, our systematic high‐resolution regional MRI analysis of a controlled pressure‐overload animal model reveals three essential findings:

1. Despite a uniform global hypertrophic stimulus, a consistent subset of animals exhibited a spatially discrete region (basolateral LV base) that failed to hypertrophy (spared hypertrophy—SH).
2. This region exhibited markedly impaired contraction (low FS), altered tissue relaxation behaviour (reduced T1, static T2) and was associated with adverse global LV remodelling (dilated EDV, reduced EF).
3. In contrast, well‐hypertrophied sectors maintained FS and displayed the expected increase in T1/T2, suggesting that hypertrophy under load remains a beneficial adaptation in these sectors. These findings therefore suggest that adaptation heterogeneity under pressure overload is not benign but rather portends regional vulnerability and eventual global dysfunction.

### 4.2 Interpretation of Regional SH and Functional Impairment

Our finding of a discrete myocardial segment that fails to hypertrophy under pressure load, and which exhibits early contractile compromise, is of substantial interest. The canonical dogma holds that in response to increased afterload the left ventricle undergoes concentric hypertrophy as a compensatory mechanism to normalize wall stress and maintain systolic performance [16]. While this is broadly valid, our results underscore that the adaptive hypertrophic response may not be uniform across the ventricle. Regional heterogeneity of hypertrophy has been reported in humans with hypertension or aortic stenosis, and our data extend this concept into the experimental pressure‐overload model [17, 18]. The absence of hypertrophy in the SH region likely reflects a local failure of myocyte growth or interstitial adaptation, which in turn predisposes to mechanical disadvantage under load and regional functional impairment. The low FS in the SH region (10.2% ± 3.1%) vs. hypertrophied regions (≈25%) illustrates this clearly. This suggests that hypertrophy may indeed serve a functional protective role in maintaining regional contractility, while failure to hypertrophy may mark a vulnerability locus [19, 20].

Mechanistically, such regional failure could reflect local differences in wall stress, fibre orientation, perfusion, metabolic capacity or microvascular reserve [19, 20]. Indeed, computational and imaging studies suggest significant spatial heterogeneity in wall stress and strain even under nominally uniform load, which may trigger divergent growth responses [1, 21, 22]. Moreover, cellular and extracellular heterogeneity (inflammation, fibroblast activation, microvascular rarefaction) may vary regionally, leading to segment‐specific trajectories of adaptation, with some regions successfully hypertrophying and others failing [22]. The present data show that the SH region did not prolong T1 or T2 (in fact T1 shortened by 5.1% ± 1.7%; *p* < 0.01), whereas hypertrophied sectors exhibited T1 increases by 22.6% ± 1.1% (*p* < 0.01). The prolonged T1 in hypertrophied tissue likely reflects myocyte hypertrophy and interstitial expansion, while in the SH region the paradoxical T1 shortening may indicate myocyte dropout, compaction of remaining tissue or early necrosis—pointing to a maladaptive rather than adaptive response [23, 24]. This interpretation is reinforced by the strong correlation between T1 shortening and reduced FS in SH sectors [24].

### 4.3 Implications for Global Ventricular Remodelling

Beyond regional findings, the presence of SH was associated with adverse global outcomes: hearts with SH exhibited significantly lower EF (32.5% ± 1.8% vs. 49.8% ± 4.1%, *p* < 0.05) and greater end‐diastolic volume, indicating dilatation and early decompensation. This suggests that regional adaptation failure may not remain isolated—rather, it may serve as a nidus for global functional decline, either by increasing wall stress elsewhere, altering loading conditions or triggering maladaptive signalling cascades [1, 4]. The notion that regional heterogeneity predisposes to global failure is supported by clinical imaging studies showing that even with preserved global ejection fraction, regional dysfunction predicts poorer outcomes [25]. Thus, our findings emphasize that uniform global hypertrophy is not the only relevant adaptation pattern; indeed, ‘non‐response’ (spared hypertrophy) may be equally predictive of adverse global remodelling.

From a translational perspective, the identification of a discrete vulnerable segment in a uniform model of afterload elevation suggests that region‐specific imaging biomarkers an regional analysis may help identify early maladaptive trajectories before global decompensation [17, 25]. The capacity of high‐resolution MRI to detect segmental phenotypic divergence underlines its potential applicability to human pressure‐overload conditions (e.g., aortic stenosis and systemic hypertension) where regional adaptation profiles may vary and influence prognosis [23, 24, 25].

### 4.4 Implications and Novel Contributions

Previous work in pressure overload models has predominantly focused on global cardiac hypertrophy, fibrosis and functional decline [5, 17, 24]. Our work adds to the literature in several important ways. Firstly, by applying a fine‐grained sectoral analysis (200 sectors) we resolve regional adaptation in high spatial resolution, a granularity seldom reported in rodent models. Secondly, by combining structural (dWT) metrics, functional (sectoral FS) and tissue‐characterisation (T1/T2 mapping) we provide insight into the link between adaptation phenotype and tissue state. Thirdly, by demonstrating regional SH as a distinct maladaptive response rather than simply a lack of hypertrophy, we extend the paradigm of adaptation failure [19, 22]. While clinical studies have observed regional intramyocardial heterogeneity of strain or shortening even in hypertrophy, to our knowledge this is the first experimental model where a discrete non‐hypertrophied region is tracked longitudinally as a predictor of global dysfunction [10, 17, 24, 25]. By doing so, our data challenge the oversimplified notion that hypertrophic remodelling is inherently detrimental and rather support the concept that appropriate hypertrophic adaptation (at regional level) may exert protective effects, whereas failure to adapt may drive worse outcomes [20].

### 4.5 Mechanistic Considerations

The mechanisms underlying regional failure to hypertrophy remain speculative, yet several possibilities merit discussion [22]. One potential factor is regional differences in mechanical burden [24, 25]. Even in a global afterload elevation model, the local distribution of wall stress depends on geometry, curvature and fibre architecture [15]. Regions with higher curvature or less favourable alignment may experience disproportionate stress and thus either failed adaptation or earlier decompensation [15, 21, 26]. Studies modelling wall stress distribution confirm significant intra‐ventricular heterogeneity [21, 26]. Furthermore, regional perfusion deficits or microvascular rarefaction may limit the hypertrophic response by restricting oxygen delivery or metabolic substrate, especially under increased workload [1, 10, 27]. This is corroborated by the known coupling of microvascular supply and hypertrophic growth [9, 10]. In parallel, regional differences in extracellular matrix remodelling may modulate myocyte growth potential [18, 19, 24]. Our T1 mapping suggests that hypertrophied sectors increased T1 but SH sectors did not, implying a suppressed or divergent fibrotic response [23, 24]. Additionally, activation of cell‐death pathways may be regionally variable under stress [10, 11, 27]. Hence, a combination of elevated mechanical load, perfusion/metabolic mismatch, microstructural matrix difference and early cell loss may contribute to regional SH and subsequent functional decline [9, 10, 11, 18, 19, 23, 24, 27]. These mechanistic hypotheses will need targeted molecular and histological verification in future work.

### 4.6 Strengths and Limitations

A major strength of this study is the integrated multiparametric MRI approach applied longitudinally with high spatial resolution, which allowed simultaneous assessment of structural, functional and tissue phenotypes. The sector‐based regional approach provided an unprecedented level of granularity in adaptation analysis. Moreover, the model was well controlled, with consistent afterload elevation and reproducible imaging conditions.

Nevertheless, several limitations warrant mention. First, the animal model—despite its strengths—may not entirely recapitulate human pressure‐overload pathophysiology, particularly in relation to chronicity and comorbidities; translational extrapolation requires caution. Second, while we identify a region of SH and associate it with functional decline, causality cannot be definitively proven in this observational design. It is possible that the region was already predisposed (e.g., by microvascular insufficiency) to adverse response. Third, histological or molecular correlates of the SH region (e.g., apoptosis, capillary density and fibroblast activation) were not exhaustively characterised in this work; such data would strengthen the link between imaging phenotype and cellular mechanism. Fourth, given that the SH phenomenon was observed in 49% of animals, there may be intrinsic biological variability or unmeasured factors (e.g., genetic background and subtle surgical variations) that determine susceptibility; further efforts to identify predictors of SH would be valuable. Fifth, although the region of interest (posterolateral basal segment) emerged reproducibly, whether other myocardial regions might exhibit similar vulnerability under different loading conditions remains to be determined.

### 4.7 Clinical and Translational Relevance

From a clinical standpoint, our findings suggest that in conditions of chronic pressure overload (such as aortic stenosis, hypertension or pressure overload in congenital heart disease), intraventricular adaptation is likely heterogeneous. Some myocardial territories may fail to hypertrophy and become loci of early contractile failure, ultimately driving global decompensation. This underscores the potential value of imaging strategies focusing on regional tissue phenotype (via parametric MRI T1/T2 or strain imaging) to identify early maladaptive responses before overt heart failure. Indeed, recent human studies using single‐cell and proteomic profiling in pressure‐overload settings demonstrate that subclinical molecular remodeling precedes overt functional decline [28, 29]. Our data therefore provide a mechanistic and imaging‐based bridge towards identifying spared hypertrophied segments as early indicators of maladaptation. Further, therapeutic strategies may need to shift from simply inhibiting hypertrophy toward supporting adaptive hypertrophy and preserving regional contractile reserve.

### 4.8 Future Directions

Building on the present findings, future research should seek to: (1) define molecular and cellular correlates of the SH region—specifically, assess microvascular density, myocyte apoptosis/necrosis, fibroblast activation and extracellular matrix organisation; (2) determine whether interventions (e.g., pharmacologic modulation of hypertrophy, angiogenesis or metabolism) can prevent the emergence of non‐hypertrophied vulnerable segments and preserve function; (3) apply similar high‐resolution regional imaging and mapping to larger animal models or human cohorts with pressure overload to validate translational relevance; (4) explore whether regional SH predicts long‐term outcomes (e.g., arrhythmia susceptibility and heart failure progression); and (5) refine imaging biomarkers (such as segmental T1/T2, strain mapping) for early identification of maladaptation in clinical practice.

## 5 Conclusions

In conclusion, our study demonstrates that under experimental pressure overload, myocardial adaptation is not uniformly hypertrophic. We identified a discrete basal posterolateral segment of the left ventricle in half of the animals which failed to hypertrophy (the SH region) and which exhibited early and pronounced contractile impairment. Importantly, this regional adaptation failure was associated with adverse global ventricular remodelling—reduced EF and dilatation. These observations indicate that not only regions of exaggerated hypertrophy but also regions of SH may drive functional decline in pressure‐overloaded ventricles. From an imaging perspective, this emphasises the need for spatially resolved phenotyping rather than simple global metrics. Translationally, our findings open a new perspective: that identifying and protecting these spared hypertrophied segments may offer a novel route to prevent progression from compensated hypertrophy to heart failure in pressure‐overload states. Ultimately, this work provides a foundation for future mechanistic, imaging and therapeutic investigations in the domain of myocardial adaptation and remodelling.

## Author Contributions

S.H. responsible for conception of study design, data acquisition and analysis, statistical assessment and writing of the manuscript. A.T.O. and V.K. involved in analysis, writing, in drafting and final approval of the manuscript submitted. V.K. and M.O. involved in data acquisition. T.V. and D.L. involved in interpretation of the data and critical review of the manuscript. V.K. and S.M. involved in analysis and interpretation of data. All authors read and approved the final manuscript.

## Funding

The authors have nothing to report.

## Ethics Statement

All animal studies have been approved by the Landesamt für Natur‐ und Umweltschutz (LANUV) Nordrhein‐Westfahlen by the registration number 84‐02.04‐2015‐A089 in accordance with the ethical standards laid down in the 1964 Declaration of Helsinki and its later amendments. Furthermore, all authors gave their informed consent prior to their inclusion in the study.

## Consent

The authors have nothing to report.

## Conflicts of Interest

The authors declare no conflicts of interest.

## Data Availability

The data that support the findings of this study are available on request from the corresponding author. The data are not publicly available due to privacy or ethical restrictions.
